# IgG4-Related Hepatic Inflammatory Pseudotumor With Extensive Portal Vein Thrombosis Mimicking Hepatocellular Carcinoma

**DOI:** 10.14309/crj.0000000000002155

**Published:** 2026-06-03

**Authors:** Mai Abouzeid, Felicita Baratelli, Mudireddy Mythri

**Affiliations:** 1 Department of Internal Medicine, North Mississippi Medical Center, Tupelo, MI; 2 Department of Pathology and Clinical Laboratories, North Mississippi Medical Center, Tupelo, MI; 3 Department of Hematology and Oncology, North Mississippi Medical Center, Tupelo, MI

**Keywords:** IgG4-related disease, hepatic inflammatory pseudotumor, portal vein thrombosis, hepaticmass, IgG4-related hepatic disease, obliterative phlebitis, storiform fibrosis, plasma cell infiltrate, hepatocellular carcinoma mimic

## Abstract

IgG4-related disease is a systemic fibroinflammatory condition that can involve any organ and frequently mimics malignancy. Hepatic manifestations are uncommon, and mass-forming lesions with portal vein involvement are exceedingly rare. We report a 72-year-old woman who presented with abdominal discomfort, anorexia, weight loss, and newly identified portal vein thrombosis in the setting of hepatic masses. Magnetic resonance imaging demonstrated a 5.6-cm heterogeneously enhancing hepatic lesion with cystic and necrotic components and thrombosis of the main and right portal veins, raising strong suspicion for hepatocellular carcinoma. Significant radiologic-pathologic discordance resulted from the initial biopsy, which only showed chronic venous outflow obstruction. The repeat biopsy revealed extensive storiform fibrosis, many IgG4-positive plasma cells, and obliterative phlebitis, supporting the diagnosis of IgG4-related hepatic inflammatory pseudotumor. Prednisone treatment (0.6 mg/kg/d) resulted in a significant regression of portal vein thrombosis and hepatic lesions, as well as a rapid clinical improvement and normalization of liver functions. This case highlights the importance of repeat tissue sampling when imaging strongly suggests malignancy, but histology remains inconclusive and underscores the dramatic steroid responsiveness of IgG4-related hepatic disease.

## Introduction

A 72-year-old woman presented with abdominal discomfort, nausea, anorexia, and bilateral visual disturbances. Laboratory evaluation revealed elevated liver enzymes and a serum IgG4 level of 6 mg/dL (reference range 2–96 mg/dL), which was within normal limits. Magnetic resonance imaging demonstrated a 5.6-cm heterogeneously enhancing hepatic lesion with cystic and necrotic components, a smaller adjacent lesion, thrombosis of the main and right portal veins, and chronic occlusion of the left portal vein. The radiographic appearance was highly concerning for hepatocellular carcinoma (HCC) with vascular invasion.

**Figure 1.**
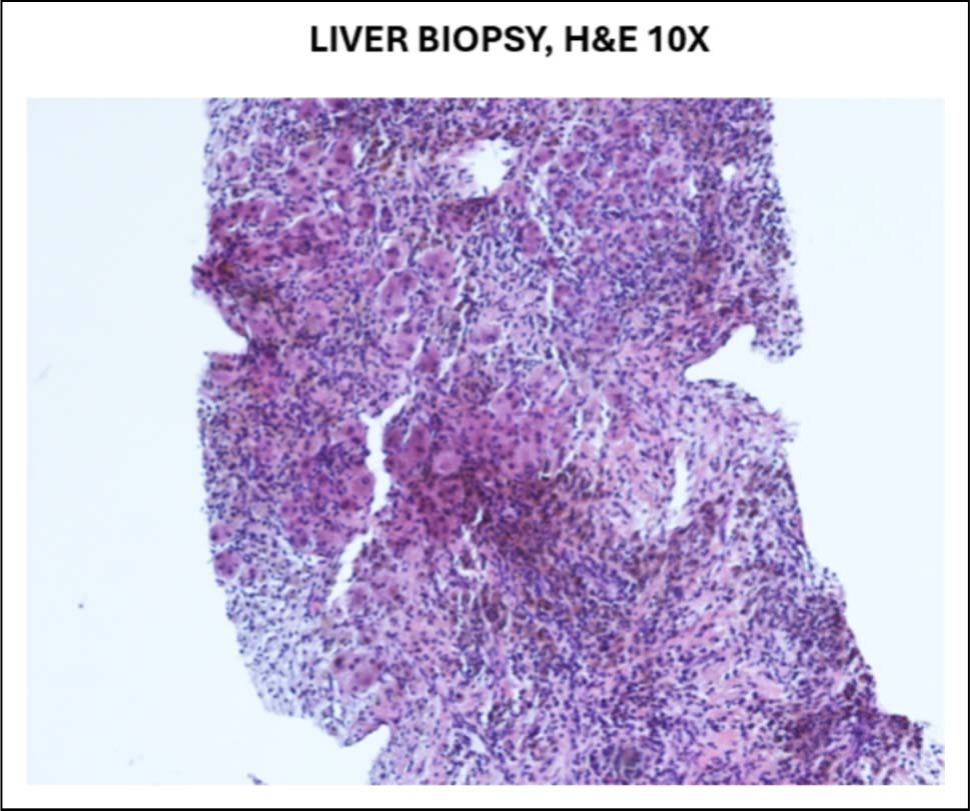
Initial liver biopsy demonstrating chronic venous outflow obstruction without evidence of malignancy on hematoxylin and eosin stain.

**Figure 2.**
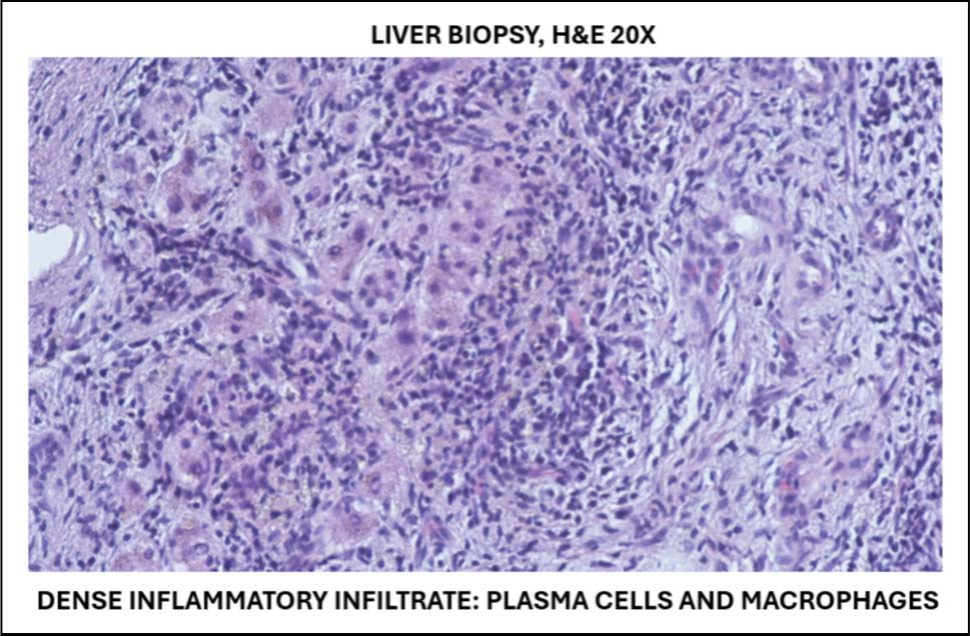
Liver biopsy, H&E 20× showing dense inflammatory infiltrate composed of plasma cells and macrophages. H&E, hematoxylin and eosin.

Initial ultrasound-guided liver biopsy showed features of chronic venous outflow obstruction without evidence of malignancy (Figure 1). Given the strong radiologic suspicion of cancer, repeat biopsy was pursued. Histopathologic examination revealed dense storiform fibrosis, abundant IgG4-positive plasma cells (>10 per high-power field with IgG4/immunoglobulin G ratio >40%), and obliterative phlebitis, confirming IgG4-related hepatic inflammatory pseudotumor (Figure 2).

The patient was treated with prednisone 0.6 mg/kg/d. Within 4–6 weeks, she experienced significant symptomatic improvement and normalization of liver enzymes. Follow-up imaging demonstrated marked regression of hepatic lesions and recanalization of portal vein thrombosis.

## DISCUSSION

IgG4-related hepatic inflammatory pseudotumor is a rare manifestation of IgG4-related disease (IgG4-RD) and frequently mimics primary hepatic malignancy. Heterogeneous enhancement, cystic or necrotic components, and vascular involvement are imaging features that often raise concern for HCC.^1–3^ Diagnostic uncertainty is heightened when portal vein thrombosis is present, as vascular invasion is typically associated with malignant tumors. Although obliterative phlebitis is a hallmark histopathologic feature of IgG4-RD, clinically apparent portal vein thrombosis detectable on imaging remains uncommon.^2,4,5^

**Figure 3.**
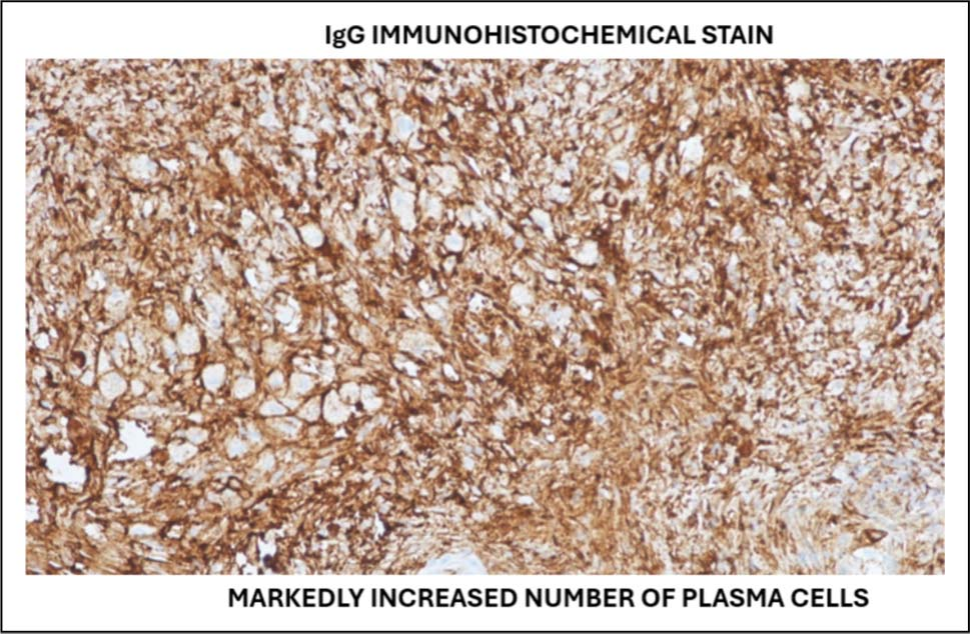
Immunoglobulin G immunohistochemical stain demonstrates markedly increased plasma cells.

Notably, this patient had a normal serum IgG4 concentration (6 mg/dL) despite biopsy-proven IgG4-related hepatic inflammatory pseudotumor (IgG4-HIPT). Approximately 20%–30% of patients with histopathologically confirmed IgG4-RD have normal serum IgG4 levels at presentation, particularly those with single-organ or limited disease.^6–8^ This case reinforces that elevated serum IgG4 is neither sensitive nor specific for diagnosis—although levels >5× the upper limit of normal have a positive predictive value of ∼75%, normal values do not exclude the disease.^9,10^ The diagnosis must rely on the integration of histopathologic findings (storiform fibrosis, IgG4+ plasma cells, obliterative phlebitis), imaging characteristics, and clinical response to therapy, as outlined in the histology, imaging, serology, other organ involvement, and response to therapy (HISORt) criteria (Figure 3).^11,12^

The portal vein thrombosis observed in this case represents a rare but well-documented manifestation of IgG4-RD's characteristic vascular pathology. Obliterative phlebitis is 1 of the 3 cardinal histopathologic features of IgG4-RD. It involves dense lymphoplasmacytic infiltration of venous walls with progressive luminal narrowing and eventual complete occlusion.^4,5,12^ The inflammatory process is driven by CD4^+^ T cells and IgG4-expressing plasmablasts that infiltrate vessel walls, triggering fibrosis through cytokine-mediated mechanisms involving interleukins 4, 10, and 13.^6,8,12^ Although obliterative phlebitis is a microscopic hallmark present in most affected tissues, clinically apparent thrombosis detectable on imaging is uncommon, making this case particularly instructive.^7,13^ The dramatic recanalization of portal vein thrombosis after corticosteroid therapy underscores that the vascular occlusion in IgG4-RD is primarily inflammatory rather than thrombotic, distinguishing it from malignancy-associated vascular invasion.^5,8^ This steroid-responsiveness of vascular lesions is a key diagnostic feature and supports the inflammatory pathogenesis of IgG4-related vascular disease.^4,12^

This case underscores the importance of recognizing radiologic-pathologic discordance. When imaging strongly suggests malignancy, but biopsy is nondiagnostic, repeat tissue sampling is essential. Sampling error is well described in hepatic lesions, particularly when tumors are heterogeneous or contain necrotic areas. IgG4-HIPT can exhibit variable histopathologic patterns, and the classic triad of dense lymphoplasmacytic infiltration, storiform fibrosis, and obliterative phlebitis may not be captured on initial biopsy.^2,5^ Given that IgG4 immunostaining is not routinely performed, clinical suspicion must guide pathologists to pursue appropriate immunohistochemistry when IgG4-RD is in the differential.^2^ As recommended in practice guidelines, persistent clinical suspicion should prompt rebiopsy to avoid misclassification of benign inflammatory disease as malignancy.^4,11^

**Figure 4.**
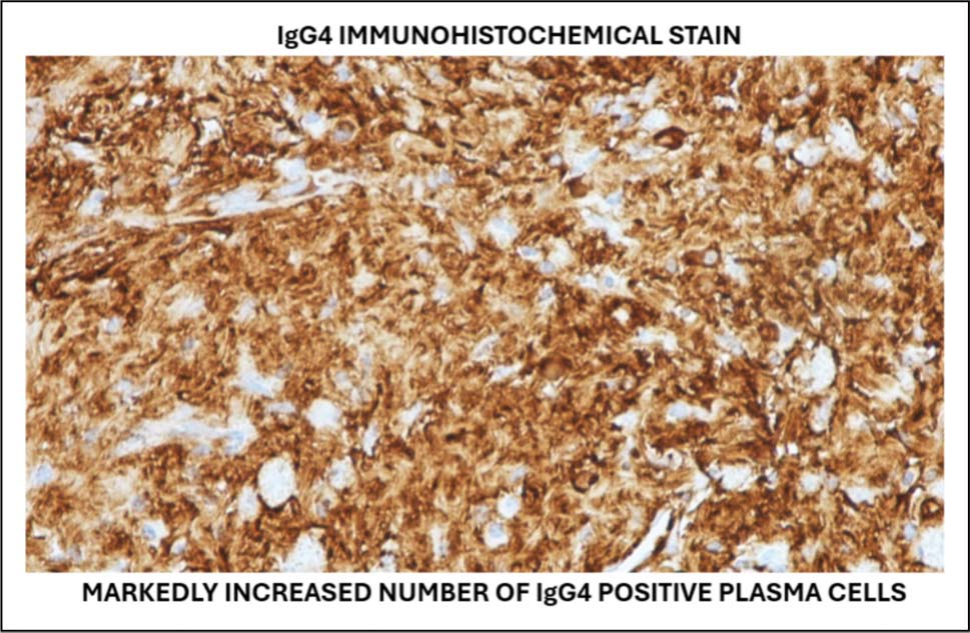
IgG4 immunohistochemical stain demonstrated markedly increased IgG4-positive plasma cells.

Definitive diagnosis requires the combination of histopathologic, serologic, radiologic, and clinical characteristics, in accordance with the 2019 American College of Rheumatology/European Alliance of Associations for Rheumatology (ACR/EULAR) classification criteria and the HISORt framework.^11,12^ The repeat biopsy in this instance revealed obliterative phlebitis and many IgG4-positive plasma cells, meeting important histologic requirements. The robust clinical and radiologic reaction to glucocorticoids further confirmed the diagnosis, as steroid responsiveness is indicative of IgG4-RD and is included in the HISORt diagnostic model (Figure 4).^11^

Glucocorticoids continue to be the primary treatment, with response rates surpassing 90%.^4,11^ Relapse occurs in 30%–50% of patients, and steroid-sparing treatments such rituximab are effective in refractory or recurrent illness.^4^ Recently, inebilizumab received FDA approval as the first drug approved exclusively for IgG4-RD, providing an alternative for individuals with relapsing or treatment-resistant conditions.^14^ Timely identification is essential, because inaccurate diagnosis may result in unwarranted oncological procedures and improper immunosuppressive treatment.

This case highlights the need to consider IgG4-HIPT in the differential diagnosis of hepatic masses with atypical imaging features, especially when vascular involvement is present, but histology is inconclusive. Awareness of this entity and adherence to a structured diagnostic approach can prevent misdiagnosis and ensure timely initiation of effective therapy.

IgG4-related hepatic inflammatory pseudotumor with portal vein thrombosis is a rare but critical mimic of HCC. Radiologic-pathologic discordance should prompt repeat biopsy to avoid misdiagnosis and unnecessary oncologic treatment. Early recognition allows effective steroid therapy and may lead to dramatic clinical and radiographic improvement.

## DISCLOSURES

Author contributions: M. Abouzeid: Conceptualization, data collection, literature review, manuscript drafting, figure preparation, and critical revision. F. Baratelli: Histopathologic interpretation, figure selection and annotation, and critical revision of the manuscript. M. Mythri: Clinical oversight, diagnostic interpretation, treatment planning, and critical manuscript review. M. Abouzeid is the article guarantor.

Financial disclosure: The authors declare no conflicts of interest.

Informed consent was obtained for this case report.

## ABBREVIATIONS:

IgG4: Immunoglobulin G subclass 4
ACR/EULAR: American College of Rheumatology/European Alliance of Associations for Rheumatology
HISORt: Histology, Imaging, Serology, Other organ involvement, and Response to therapy
